# Comparative analysis of the morphological and biomechanical properties of normal cornea and keratoconus at different stages

**DOI:** 10.1007/s10792-021-01929-4

**Published:** 2021-07-07

**Authors:** Ying Wu, Li-Li Guo, Lei Tian, Ze-Quan Xu, Qian Li, Jian Hu, Yi-Fei Huang, Li-Qiang Wang

**Affiliations:** 1 Department of Ophthalmology, Chinese People’s Liberation Army General Hospital, Beijing, 100853 China; 2 Beijing Institute of Ophthalmology, Beijing Tongren Eye Center, Beijing Tongren Hospital, Capital Medical University, Beijing Ophthalmology & Visual Sciences Key Laboratory, Beijing, 100730 China; 3 Beijing Advanced Innovation Center for Big Data-Based Precision Medicine, Beihang University and Capital Medical University, Beijing Tongren Hospital, Beijing, 100730 China

**Keywords:** Keratoconus, Corneal morphology, Corneal biomechanics, Corneal visualization Scheimpflug technology

## Abstract

**Purpose:**

To compare the morphological and biomechanical properties of normal cornea and keratoconus at different stages.

**Methods:**

A total of 408 patients (517 eyes) with keratoconus were included in this study. According to the Topographic Keratoconus (TKC) grading method, keratoconus was divided into stage I (TKC = 1, 130 eyes), stage II (TKC = 1–2, 2, 164 eyes), stage III (TKC = 2–3, 3, 125 eyes) and stage IV (TKC = 3–4, 4, 98 eyes). A total of 158 normal subjects (158 eyes) were recruited as the normal group. The corneal morphological parameters and biomechanical parameters were obtained with Scheimpflug tomography (Pentacam) and corneal visualization Scheimpflug technology (Corvis ST), and the receiver operating characteristic (ROC) curves were drawn.

**Results:**

Each corneal morphological and most biomechanical parameters of the keratoconic eyes were significantly different from those of the normal eyes in this study (*p* < 0.001). ROC curve demonstrated that most parameters in this study showed high efficiency in diagnosing keratoconus (the area under the ROC (AUC) was > 0.9), with the Belin-Ambrósio deviation (BAD-D) and Tomographic and Biomechanical Index (TBI) showing higher efficiency. The efficiency of BAD-D and TBI was high in differentiating keratoconus at different stages (AUC > 0.963). The comparison of ROC curves of keratoconus at different stages did not reveal statistically significant differences for TBI.

**Conclusion:**

BAD-D and TBI can effectively diagnose stage I keratoconus. Moreover, the efficiency of TBI is the same in diagnosing keratoconus at all stages, while the diagnostic efficiency of other parameters increases with the increase in keratoconus stages.

## Introduction

Keratoconus is a chronic progressive disease, characterized by thinning of the central or para-central corneal stroma and protruding conical shape [1]. The majority of keratoconus patients are adolescents, and 90% of cases are binocular, often with asymmetric progression in both eyes [2, 3]. During keratoconus progression, the Bowman's membrane breaks, leading to the development of disproportion in the compositions of collagen fibers. The increase in protein kinase and other catabolic enzymes and the decrease in protein kinase inhibitor in cornea lead to the destruction of corneal stroma structure, causing instability of corneal biomechanical properties and the weakening of mechanical strength [4, 5]. Therefore, the diagnosis of keratoconus by analyzing corneal biomechanical properties has become a research hotspot.

The ocular response analyzer (ORA) [6] and the corneal visualization Scheimpflug technology (Corvis ST) [7] are the only in vivo corneal biomechanical measurement equipment currently used in clinical practice. Both devices utilize the reaction of cornea under the action of fast air pulse to indirectly evaluate the biomechanical properties of cornea [8]. Although studies have reported that the corneal hysteresis (CH) and corneal resistance factor (CRF) of typical keratoconus measured by ORA are lower than those of normal cornea, their distributions mostly overlap; thus, their sensitivity and specificity in diagnosing keratoconus are relatively low [9]. And the limited corneal detection area of ORA results in poor diagnostic ability of CH and CRF in normal cornea and early keratoconus. The biomechanical parameters measured with early version of Corvis ST (1.00r30) have been proven to be significantly different between keratoconus and normal cornea. Among them, the maximum compression depth (DA) is efficient in diagnosing keratoconus, but could be easily affected by intraocular pressure (IOP); thus, its efficiency in diagnosing mild keratoconus is low [10].

With the update of Corvis ST software (1.5r1902), some new parameters have been incorporated [11], including the maximum value of the ratio between the deformation amplitude at the apex and at 2 mm from the central apex (DA Ratio 2), Ambrósio related thickness to the horizontal profile (ARTh), inverse of the radius of curvature during the concave phase of the deformation (Integrated Radius), stiffness parameter at first application (SPA1) and Corvis biomechanical index (CBI), as well as Tomographic and Biomechanical Index (TBI), which was obtained with combined use of Pentacam and Corvis ST. Studies have shown that these new parameters are highly efficient in diagnosing keratoconus [12–14]. However, there are few studies on the combined use of Pentacam and Corvis ST for keratoconus at different stages.

The purpose of this study is to measure and compare the morphological and biomechanical properties of normal cornea and keratoconus at different stages. Moreover, the efficiency of corneal morphological and biomechanical parameters in diagnosing keratoconus at different stages was explored and compared the diagnostic efficacy among new parameters.

## Patients and methods

*Study group* A total of 408 keratoconus patients (517 eyes) and 158 normal subjects (158 eyes) who visited Chinese PLA General Hospital and Beijing Tongren Hospital from January 2018 to December 2019 were recruited. Given that most keratoconus cases develop binocularly and progress asymmetrically, patients diagnosed as binocular keratoconus were included in this study, and the right eye of all normal subjects were included. According to the Topographic Keratoconus (TKC) grading method, keratoconus cases were divided into stage I (130 eyes), stage II (164 eyes), stage III (125 eyes) and stage IV (98 eyes). The study protocol was approved by the Chinese PLA General Hospital, Beijing, China and the ethics committee of Beijing Tongren Hospital, Capital Medical University. Informed consent was obtained from all participants.

*Inclusion criteria* Keratoconus group: the diagnosis of keratoconus was based on the Rabinowitz criteria [15]. 1. A history of myopia and astigmatism, decreased vision, corrected visual acuity < 1.0; at least one of the following signs was positive in slit lamp examination: corneal stromal thinning, conical corneal protrusion, Fleischer ring, Vogt's striae, epithelial or subepithelial scar; corneal topography showed that diopter was > 47D in the central area of anterior corneal surface; the diopter difference between areas 3 mm below and above the central corneal area was > 3D; the difference in diopter of anterior surface of central corneal area between right and left eyes was > 1D. 2. The central corneal diopter was > 46.5D, the diopter difference between areas 3 mm above and below was > 1.26D, and the corneal diopter difference between right and left eyes of the same patient was > 0.92D. Keratoconus grading method [16]: Based on the TKC grading method provided by Pentacam, keratoconus patients were divided into stage I (TKC = 1) group, stage II (TKC = 1–2, 2) group, stage III (TKC = 2–3, 3) group and stage IV (TKC = 3–4, 4) group. Normal group: subjects were matched based on age and gender of patients in the keratoconus group. The inclusion criteria of the normal group were: binocular anterior surface curvature < 46.5D; posterior surface curvature < 57.2D; thinnest corneal thickness (TCT) > 490 μm [17].

*Exclusion criteria* Patients with the following conditions were excluded: other ocular diseases, trauma, surgery history, systemic diseases that could affect the eyes; patients who wore soft contact lens should have stopped wearing them for > 2 weeks; patients who wore hard contact lens should have stopped wearing them for > 1 month.

*Examinations* All the subjects received routine ophthalmic examinations, including uncorrected distant vision, slit lamp microscopy and fundus examination. Additionally, corneal morphological parameters were obtained with Pentacam and biomechanical parameters were obtained with Corvis ST.

Pentacam (Oculus Optikgeräte GmbH, Wetzlar, Germany, software version 1.20r134) is a commonly used corneal tomography image analysis instrument. It uses Scheimpflug camera to scan from the anterior surface of cornea to the posterior surface of lens and obtains the morphological parameters of anterior segment by calculating and analyzing the data collected.

Corvis ST (Oculus Optikgeräte GmbH, Wetzlar, Germany, software version 1.5r1902) records the compression deformation of cornea caused by the air pulse with a high-speed Scheimpflug-camera. The bi-directional applanation and maximum compression status during the deformation are specially monitored and analyzed, so that the parameters reflecting the corneal biomechanical characteristics can be obtained.

Pentacam and Corvis ST were operated by the same trained and skilled technicians. The display of "OK" indicated good measurement quality, and the record data were saved. Table1 shows the parameters of Pentacam and Corvis ST included in this study.

**Table 1:** Corneal morphological and biomechanical parameters obtained with Pentacam and Corvis ST

Parameters		Parameters	
*Pentacam*		A2V	The second velocity of applanation
K1	Steepest keratometric reading	HCT	Time from the start until the highest concavity
K2	Flattest keratometric reading	PD	Peak distance
Km F	Mean keratometry from the anterior corneal surface	Radius	Central curvature radius at the highest concavity
Kmax	Maximum keratometry from the anterior corneal surface	A1DfL	Deflection length of the first applanation
Astig F	Central astigmatism from the anterior corneal surface	HC DfL	Deflection length of the highest concavity
CCT	Central corneal thickness	A2 DfL	Deflection length of the second applanation
TP	Pachymetry at the thinnest point	A1 DfA	Deflection amplitude of the first applanation
ISV	Index of surface variance	HC DfA	Deflection amplitude of the highest concavity
IVA	Index of vertical asymmetry	A2 DfA	Deflection amplitude of the second applanation
KI	Keratoconus index	DA Ratio 2	The maximal value of the ratio between the deformation amplitude at the apex and at 2 mm from the corneal apex
CKI	Central keratoconus index	DA Ratio 1	The maximal value of the ratio between the deformation amplitude at the apex and at 1 mm from the corneal apex
IHA	Index of height asymmetry	ARTh	Ambrósio relational thickness to the horizontal profile
IHD	Index of height decentration	bIOP	Biomechanical-corrected intraocular pressure
BAD-D	Belin-Ambrósio deviation	Integrated Radius	Inverse of the radius of curvature during the concave phase of the deformation
*Corvis ST*			
A1T	First applanation time	SPA1	Stiffness parameter at the first applanation
A1V	First velocity of applanation	CBI	Corvis biomechanical index
A2T	Second applanation time	TBI	Tomographic and Biomechanical Index

*Analysis index* (Table1).

*Statistical methods* Statistical analysis and drawing were completed with R language.

4.0.5(https://www.r-project.org/) and SPSS 20 (IBM Corp., Armonk, NY, USA). Shapiro test was used to test the normality of corneal morphological and biomechanical indexes; the mean ± standard deviation was adopted to describe the normal distribution of measurement data; the median and quartile were used to describe the non-normal data. Parameters of keratoconus at different stages and normal cornea were compared between the keratoconus group and the normal group with Kruskal–Wallis test, with *p* < 0.05 considered as statistically significant. Pairwise comparison was conducted with post-hoc test, and as multiple comparisons were made, the test level was corrected to 0.017 according to Bonferroni principle (p < 0.017 was considered as statistically significant). Receiver operating characteristic (ROC) curve was used to explore the diagnostic ability of corneal morphological and biomechanical parameters for distinguishing keratoconus at different stages and normal cornea. Delong test was used to compare the areas under curves (AUCs) of different parameters and AUCs of the same parameter in keratoconus at different stages. In the pairwise comparison of ROC curve, the test level was corrected to 0.0023 and 0.005 according to Bonferroni principle.

## Results

There were 408 cases (517 eyes) in the keratoconus group (289 eyes from males and 228 eyes from females), with an average age of (22.56 ± 7.77) years (range: 13–45 years). There were 158 cases (158 eyes) in the normal group (99 males and 59 females), with an average age of (23.08 ± 4.61) years (range: 16–40 years). There were 130 eyes with stage I keratoconus, with an average age of (20.53 ± 8.50) years (range: 13–39 years), 164 eyes with stage II keratoconus, with an average age of (23.04 ± 8.2) years (range: 13–45 years), 125 eyes with stage III keratoconus, with an average age of (23.89 ± 6.73) years (range: 15–32 years), and 98 eyes with stage IV keratoconus, with an average age of (22.78 ± 6.76) years (range: 16–36 years). There was no significant difference in age between the keratoconus group and the normal group (p = 0.056). There was no significant difference in gender between the keratoconus group and the normal group (p = 0.133, chi-square test).

*Comparison between groups* Among the corneal morphological and biomechanical parameters, except HCT and A2 DFL, there were significant differences in all other parameters among the six groups (all *p* < 0.001), as shown in Table 2. Significant differences were found in all other parameters between the normal group and the keratoconus group (all *p* < 0.017), except HCT, A1 DfL and A2 DfL. Significant differences were found in all other parameters between the normal group and the stage I keratoconus group (all *p* < 0.017), except HCT, PD, A1 DfL, A2 DfL, HC DfL and A2 DfA. Significant differences were found in all other parameters between the normal group and the stage II keratoconus group (all *p* < 0.017), except HCT, A1 DfL, A2 DfL and HC DfL. Significant differences were found in all other parameters between the normal group and the stage III and IV keratoconus groups (all *p* < 0.017), except HCT and A2 DfL.

**Table 2:** Differences in corneal morphological and biomechanical parameters between normal group and keratoconus group as well as KC at different stages. (skewed distribution, median and range of variation)

	NL group	KC				KC group	*p*
		Stage I KC	Stage II KC	Stage III KC	Stage IV KC		
K1	42.40(41.50–43.30)	43.35(42.10–44.30)	44.70(42.80–46.65)	48.10(45.80–50.20)	56.65(51.00–60.30)	45.60(43.30–49.30)	< 0.001
K2	43.95(43.00–44.90)	45.85(44.70–47.20)	48.00(45.60–49.80)	52.60(50.30–55.30)	60.35(55.40–65.30)	48.90(46.30–53.80)	< 0.001
Km	43.20(42.40–44.00)	44.65(43.30–45.70)	46.20(44.20–48.10)	50.00(48.30–52.60)	58.75(53.80–62.80)	47.20(44.60–51.30)	< 0.001
Kmax	44.40(43.70–45.60)	47.65(45.70–48.90)	52.25(50.05–54.65)	60.90(57.90–63.30)	73.00(67.30–80.00)	54.00(49.10–62.30)	< 0.001
Astig F	1.20(0.80–1.90)	2.60(1.50–3.40)	3.05(2.00–4.45)	4.20(2.90–6.00)	4.00(2.20–6.60)	3.30(2.00–4.80)	< 0.001
CCT	553.59(537.63–578.00)	510.50(483.00–531.00)	477.43(457.50–502.00)	449.00(432.00–472.00)	430.00(400.00–457.00)	472.00(439.00–504.00)	< 0.001
TP	548.00(533.00–573.00)	500.50(478.00–522.00)	467.50(449.50–487.50)	441.00(419.00–460.00)	418.00(388.00–441.00)	461.00(429.00–493.00)	< 0.001
ISV	17.00(14.00–22.00)	37.00(33.00–41.00)	62.50(52.00–74.00)	101.00(94.00–112.00)	156.50(142.00–175.00)	75.00(46.00–112.00)	< 0.001
IVA	0.11(0.08–0.15)	0.30(0.23–0.38)	0.63(0.49–0.77)	1.00(0.79–1.20)	1.26(0.99–1.62)	0.68(0.39–1.02)	< 0.001
KI	1.03(1.01–1.04)	1.09(1.07–1.10)	1.15(1.11–1.19)	1.26(1.22–1.30)	1.40(1.33–1.49)	1.17(1.10–1.28)	< 0.001
CKI	1.01(1.00–1.01)	1.02(1.01–1.02)	1.04(1.02–1.07)	1.10(1.07–1.14)	1.19(1.15–1.25)	1.05(1.02–1.12)	< 0.001
IHA	5.40(2.10–9.70)	19.60(9.80–26.50)	19.15(9.10–41.05)	27.60(15.60–52.80)	31.15(16.60–49.50)	22.20(12.50–38.90)	< 0.001
IHD	0.01(0.01–0.01)	0.03(0.02–0.04)	0.08(0.06–0.10)	0.15(0.12–0.17)	0.23(0.18–0.27)	0.10(0.05–0.16)	< 0.001
BAD	0.96(0.53–1.29)	3.01(2.15–4.10)	6.38(5.28–7.90)	10.77(9.49–12.26)	17.42(14.11–21.35)	7.58(4.49–11.79)	< 0.001
A1T	7.40(7.24–7.59)	7.19(7.01–7.33)	7.02(6.87–7.17)	6.96(6.79–7.13)	6.92(6.73–7.08)	7.02(6.87–7.21)	< 0.001
A1V	0.15(0.13–0.16)	0.16(0.15–0.17)	0.17(0.15–0.18)	0.18(0.16–0.20)	0.19(0.17–0.21)	0.17(0.16–0.19)	< 0.001
A2T	21.81(21.52–22.04)	21.96(21.73–22.19)	22.07(21.85–22.35)	22.16(21.90–22.37)	22.22(21.86–22.42)	22.12(21.83–22.35)	< 0.001
A2V	−0.27(−0.29–0.25)	−0.29(−0.32–0.26)	−0.32(−0.34–0.28)	−0.34(−0.37–0.31)	−0.38(−0.44–0.32)	−0.32(−0.36–0.28)	< 0.001
HCT	16.86(16.63–17.13)	16.86(16.63–17.33)	16.86(16.63–17.19)	16.86(16.40–17.09)	16.86(16.63–17.09)	16.86(16.63–17.09)	0.763
PD	5.17(4.96–5.28)	5.14(5.01–5.35)	5.23(5.03–5.40)	5.24(5.13–5.38)	5.24(5.07–5.41)	5.21(5.06–5.38)	< 0.001
Radius	7.31(6.95–7.94)	6.44(5.83–7.02)	5.65(5.06–6.35)	5.27(4.73–5.70)	4.38(3.82–5.16)	5.55(4.84–6.42)	< 0.001
A1 DfL	2.33(2.22–2.42)	2.31(2.20–2.37)	2.35(2.23–2.43)	2.41(2.25–2.52)	2.48(2.33–2.63)	2.36(2.24–2.48)	< 0.001
HC DfL	6.70(6.34–6.96)	6.60(6.28–6.85)	6.67(6.20–6.96)	6.53(6.25–6.78)	6.29(5.90–6.63)	6.54(6.18–6.83)	< 0.001
A2 DfL	2.80(2.56–3.42)	2.78(2.37–3.48)	2.85(2.45–3.52)	2.81(2.48–3.62)	3.01(2.48–3.64)	2.86(2.44–3.55)	0.353
A1 DfA	0.10(0.09–0.10)	0.10(0.09–0.10)	0.10(0.10–0.11)	0.11(0.10–0.12)	0.13(0.12–0.14)	0.11(0.10–0.12)	< 0.001
HC DfA	0.91(0.85–0.97)	0.96(0.89–1.04)	1.04(0.96–1.11)	1.12(1.04–1.20)	1.22(1.12–1.35)	1.07(0.97–1.18)	< 0.001
A2 DfA	0.11(0.10–0.12)	0.11(0.10–0.12)	0.11(0.10–0.13)	0.13(0.11–0.14)	0.15(0.13–0.17)	0.12(0.11–0.14)	< 0.001
DA Ratio 2	4.06(3.85–4.30)	4.62(4.34–5.29)	5.31(4.76–6.01)	6.13(5.70–6.80)	7.25(6.10–8.81)	5.67(4.79–6.52)	< 0.001
DA Ratio 1	1.54(1.52–1.58)	1.60(1.57–1.66)	1.64(1.61–1.69)	1.71(1.66–1.76)	1.78(1.68–1.83)	1.67(1.60–1.74)	< 0.001
ARTh	452.06(390.36–524.38)	362.12(293.19–475.31)	229.31(181.91–284.65)	157.87(125.12–206.00)	103.27(79.78–147.78)	212.10(139.63–303.59)	< 0.001
bIOP	14.90(13.70–15.82)	14.70(13.20–15.90)	13.80(12.60–15.20)	13.90(12.50–15.20)	13.70(12.80–14.70)	14.05(12.70–15.30)	< 0.001
Integrated Radius	7.75(7.31–8.35)	9.33(8.44–10.42)	10.90(9.74–12.39)	12.41(11.30–13.74)	14.82(12.08–17.55)	11.31(9.56–13.24)	< 0.001
SPA1	107.21(96.17–117.78)	80.64(68.54–98.19)	63.45(51.04–73.65)	48.13(42.24–58.98)	38.45(28.48–48.23)	58.84(43.91–75.02)	< 0.001
CBI	0.01(0.00–0.07)	0.69(0.01–0.99)	1.00(0.97–1.00)	1.00(1.00–1.00)	1.00(1.00–1.00)	1.00(0.96–1.00)	< 0.001
TBI	0.14(0.02–0.26)	1.00(0.98–1.00)	1.00(1.00–1.00)	1.00(1.00–1.00)	1.00(1.00–1.00)	1.00(1.00–1.00)	< 0.001

*KC* keratoconus, *NL* normal cornea

**Fig. 1:**
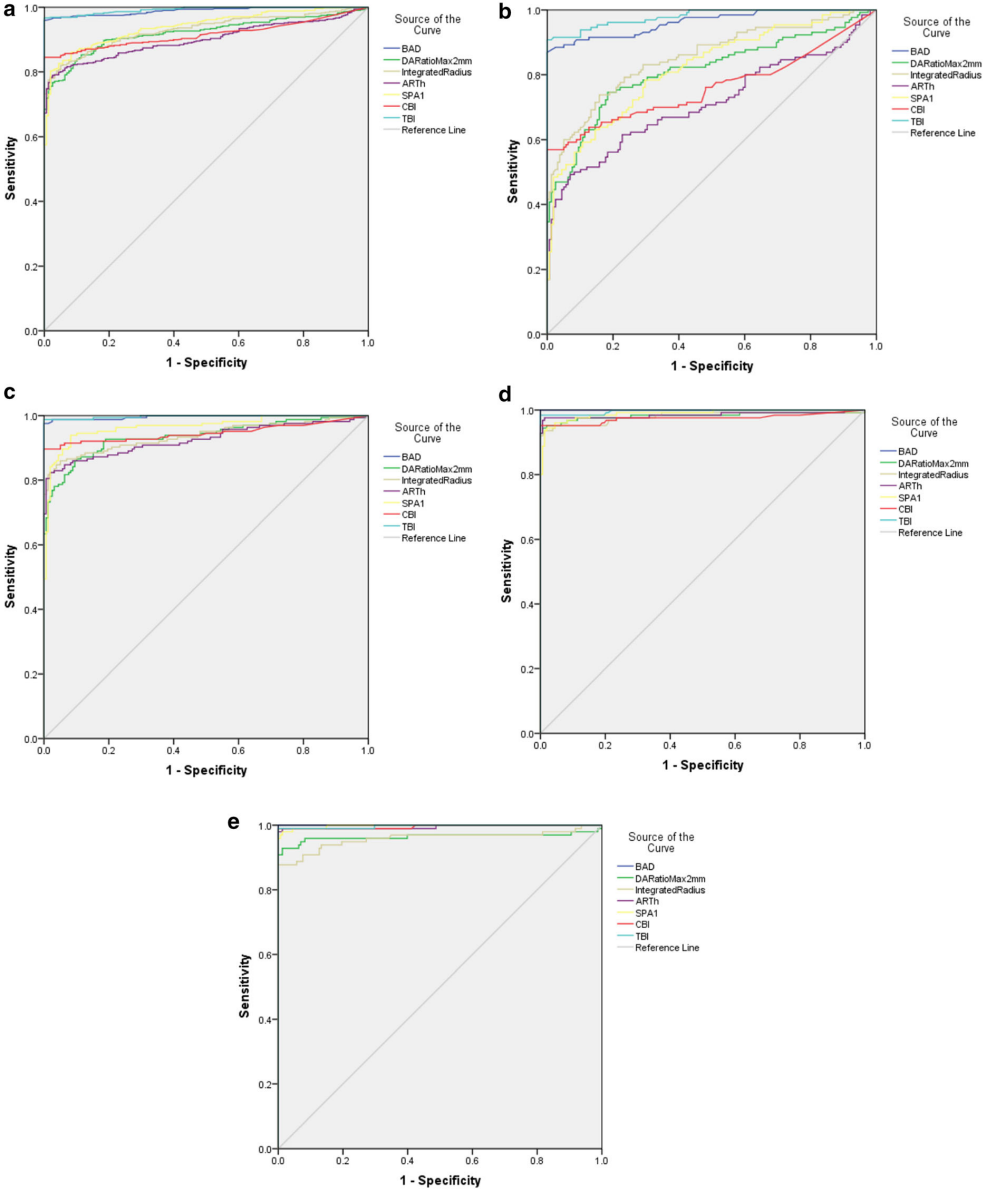
ROC curves of the different groups **a** NL group vs. KC group, **b** NL group vs. stage I KC group, **c** NL group vs. stage II KC group, **d** NL group vs. stage III KC group, **e** NL group vs. stage IV KC group

*ROC curve analysis* ROC curve analysis showed that the area under the ROC curve (AUC) of BAD-D, DA Ratio 2, ARTh, Integrated Radius, SPA1, CBI and TBI in diagnosing keratoconus were all > 0.9 (Table 3). ROC curve analysis on normal cornea and keratoconus at different stages showed that the AUCs of BAD-D and TBI in distinguishing normal cornea from keratoconus at different stages were all > 0.96. The efficiency of DA Ratio 2, ARTh, Integrated Radius, SPA1 and CBI in diagnosing stage I keratoconus was relatively low, but the AUCs of all parameters increased with the increase in keratoconus stages. Figure 1 a-e shows the ROC curves of all parameters in diagnosing keratoconus, and keratoconus at different stages.

**Table 3:** ROC curve analysis of normal group and keratoconus at different stages

Parameters	AUC (95% CI)	Cutoff	Sensitivity, %	Specificity, %
*KC group vs. NL group*				
BAD-D	0.989	1.595	0.959	1
DA Ratio 2	0.921	4.742	0.768	0.975
ARTh	0.9	328.6	0.783	0.981
Integrated Radius	0.93	9.024	0.82	0.949
SP-A1	0.94	79.13	0.802	0.981
CBI	0.916	0.516	0.845	1
TBI	0.993	0.515	0.967	1
*Stage I KC group vs. NL group*				
BAD-D	0.963	1.595	1	1
DA Ratio 2	0.811	4.35	0.746	0.816
ARTh	0.712	356.9	0.492	0.93
Integrated Radius	0.853	8.573	0.738	0.842
SPA1	0.818	98.75	0.785	0.703
CBI	0.761	0.516	0.569	1
TBI	0.979	0.515	0.967	1
*Stage II KC group vs. NL group*				
BAD-D	0.996	1.67	0.976	1
DA Ratio 2	0.935	4.523	0.86	0.905
ARTh	0.926	328.6	0.823	0.981
Integrated Radius	0.937	9.18	0.848	0.962
SPA1	0.965	85.66	0.939	0.918
CBI	0.946	0.516	0.896	1
TBI	0.997	0.574	0.988	1
*Stage III KC group vs. NL group*				
BAD-D	1	3.02	1	1
DA Ratio 2	0.983	4.903	0.944	0.994
ARTh	0.985	313.4	0.976	0.987
Integrated Radius	0.980	9.423	0.936	0.987
SPA1	0.989	77.22	0.944	0.981
CBI	0.975	0.718	0.952	1
TBI	0.997	0.71	0.984	1
*Stage IV KC group vs. NL group*				
BAD-D	1	3.32	1	1
DA Ratio 2	0.964	4.806	0.86	0.905
ARTh	0.995	258.1	0.823	0.981
Integrated Radius	0.958	9.696	0.848	0.962
SPA1	0.998	72.27	0.939	0.918
CBI	0.996	0.701	0.896	1
TBI	0.997	0.566	0.988	1

*KC* keratoconus, *NL* normal cornea, *AUC* area under the receiver operating characteristic curve

*Comparison of ROC curves of different parameters in keratoconus at the same stage* Comparison of the ROC curves between the normal group and the keratoconus group showed that the ROC curves of both BAD-D and TBI were statistically different from those of DA Ratio 2, Integrated Radius, SPA1 and CBI. In addition, the AUCs of BAD-D and TBI were greater than those of other parameters. Therefore, the diagnostic efficiency of BAD-D and TBI were higher than that of other parameters. However, no significant difference was found in the AUCs of BAD-D and TBI (*p* = 0.232). Hence, efficiency of BAD-D and TBI was the highest among all parameters in diagnosing keratoconus. The ROC curves of the normal group and the stage I keratoconus group showed that BAD-D and TBI were the most effective among the seven parameters, while ARTh and CBI showed the lowest efficiency. The ROC curves of the normal group and the stage II keratoconus group showed that BAD-D and TBI were the most effective among the seven parameters, while the other parameters showed the same efficiency in the diagnosis of stage II keratoconus. The ROC curves of the normal group and the stage III and IV keratoconus groups showed no significant difference among the seven parameters in diagnosing keratoconus, indicating the same diagnostic efficiency (Table 4).

**Table 4:** Comparison of ROC curves of different parameters among groups

Two curves under comparison		D 0	P 0	D 1	P 1	D 2	P 2	D 3	P 3	D 4	P 4
BAD-D	DA Ratio 2	7.164	< 0.001	6.073	< 0.001	4.425	< 0.001	1.803	0.071	2.080	0.037
	ARTh	8.381	< 0.001	8.849	< 0.001	4.494	< 0.001	1.649	0.098	1.003	0.316
	Integrate	6.602	< 0.001	4.976	< 0.001	4.170	< 0.001	2.094	0.036	2.534	0.011
	SP-A1	5.973	< 0.001	6.029	< 0.001	3.170	0.0015	2.087	0.036	1.327	0.185
	CBI	7.478	< 0.001	7.030	< 0.001	3.495	< 0.001	2.106	0.035	0.996	0.319
	TBI	− 1.194	0.232	− 1.803	0.071	− 0.518	0.604	1.386	0.166	0.993	0.321
DA Ratio 2	ARTh	2.014	0.044	3.238	0.001	0.672	0.501	− 0.274	0.784	− 1.740	0.082
	Integrate	− 1.206	0.228	− 2.157	0.031	− 0.118	0.905	0.521	0.602	0.334	0.738
	SP-A1	− 2.254	0.024	− 0.301	0.763	− 0.301	0.763	− 1.126	0.260	− 2.009	0.044
	CBI	0.528	0.597	2.005	0.045	− 2.843	0.005	0.650	0.516	− 1.775	0.075
	TBI	− 7.325	< 0.001	− 6.527	< 0.001	− 4.524	< 0.001	− 1.620	0.105	− 1.872	0.061
ARTh	Integrate	− 2.629	0.009	− 4.732	< 0.001	− 0.688	0.491	0.450	0.652	2.130	0.033
	SP-A1	− 2.311	0.021	− 3.539	< 0.001	− 2.848	0.044	− 0.458	0.646	− 0.827	0.408
	CBI	− 2.392	0.017	2.547	0.011	− 1.784	0.074	0.981	0.326	− 1.009	0.313
	TBI	− 8.549	< 0.001	− 8.766	< 0.001	− 4.589	< 0.001	− 1.682	0.092	− 0.985	0.324
Integrate	SP-A1	− 1.113	0.266	1.471	0.141	− 2.130	0.033	− 1.458	0.145	− 2.460	0.014
	CBI	1.342	0.180	3.271	0.001	− 0.590	0.554	0.404	0.686	− 2.232	0.026
	TBI	− 6.801	< 0.001	− 5.538	< 0.001	− 4.294	< 0.001	− 1.836	0.066	− 2.331	0.019
SP-A1	CBI	2.983	0.003	2.389	0.017	1.770	0.076	1.358	0.175	0.717	0.472
	TBI	− 6.506	< 0.001	− 6.843	< 0.001	− 3.273	0.001	− 1.489	0.136	0.517	0.605
CBI	TBI	− 7.610	< 0.001	− 7.339	< 0.001	− 3.592	< 0.001	− 1.998	0.046	− 0.923	0.355

D: Statistics of Delong test; 0: NL group vs. KC group; 1. NL group vs. stage I KC group; 2: NL group vs. stage II KC group; 3: NL group vs. stage III KC group; 4: NL group vs. stage IV KC group

Due to multiple comparisons, the test level was corrected according to Bonferroni principle, and *p* < 0.0023 was considered as statistically significant

*Comparison of ROC curves of the same parameter in keratoconus at different stages* There was no significant difference in the ROC curves of TBI in keratoconus at different stages, and no significant difference was found in pairwise comparison, which indicated that TBI had the same efficiency in diagnosing keratoconus at all stages. Moreover, statistically significant difference was found in the ROC curves of the other six parameters for diagnosing stage I keratoconus and keratoconus at other stages, indicating that these parameters had lower ability in diagnosing stage I keratoconus than keratoconus at other stages (Table 5).

**Table 5:** Comparison of ROC curves of the same parameter in diagnosing keratoconus at different stages

parameter	D0	*P*0	D1	*P*1	D2	*P*2	D3	*P*3	D4	*P*4	D5	*P*5
BAD-D	− 2.989	0.003	− 3.401	< 0.001	− 3.401	< 0.001	− 1.468	0.138	− 1.468	0.138	NA	NA
DA Ratio 2	− 4.509	< 0.001	− 5.965	< 0.001	− 4.756	< 0.001	− 2.731	0.006	− 1.272	0.204	0.936	0.35
ARTh	− 5.902	< 0.001	− 8.1039	< 0.001	− 8.620	< 0.001	− 3.194	0.0015	− 4.008	< 0.001	− 0.953	0.341
Integrated Radius	− 3.069	0.002	− 5.087	< 0.001	− 3.718	< 0.001	− 2.489	0.013	− 0.981	0.327	1.148	0.251
SP-A1	− 5.458	< 0.001	− 6.670	< 0.001	− 7.144	< 0.001	− 2.147	0.032	− 3.271	< 0.001	− 1.603	0.110
CBI	− 5.343	< 0.001	− 6.339	< 0.001	− 7.435	< 0.001	− 1.515	0.13	− 3.192	0.0015	− 1.642	0.102
TBI	− 2.389	0.017	− 2.282	0.023	− 2.249	0.025	0.182	0.855	0.08	0.936	− 0.072	0.94

D: Statistics of Delong test; 0: stage I KC group vs. stage II KC group; 1: stage I KC group vs. stage III KC group; 2: stage I KC group vs. stage IV KC group; 3: stage II KC group vs. stage III KC group; 4: stage II KC group vs. stage IV KC group; 5: stage III KC group vs. stage IV KC group

Due to multiple comparisons, the test level was corrected according to Bonferroni principle, and *p* < 0.005 was considered as statistically significant

## Discussion

Keratoconus is a type of corneal ectatic disorder, which can lead to serious decline of corneal optical performance and gradual decrease in asymmetric binocular vision. As the absolute contraindication of keratorefractive surgery, its exact preoperative diagnosis is especially important. Despite the fact that the clinical diagnosis of moderate and advanced keratoconus is not difficult, the diagnosis of early keratoconus still remains a challenge [18]. Early keratoconus is considered as one of the most important independent risk factors for iatrogenic keratoectasis. At present, the main auxiliary diagnostic methods for the diagnosis of keratoconus are corneal topography, but this method has certain limitations for the diagnosis of early keratoconus [19, 20]. Studies have shown that the changes of corneal biomechanical characteristics may occur before the typical corneal morphological changes [5, 21]. In this study, the morphological and biomechanical characteristics of normal cornea and keratoconus at different stages were analyzed, as well as the diagnostic efficiency of different parameters in the diagnosis of keratoconus at different stages.

Several studies have shown that there are significant differences in corneal morphological parameters between the normal cornea and keratoconus [16, 22]. This study also showed significant differences in all corneal morphological parameters between the normal cornea and keratoconus at different stages. For instance, the corneal curvature, ISV, IVA, KI, CKI, IHA, and IHD of the keratoconus group were significantly higher than those of the normal group, but the thickness was significantly less than that of normal group.

Numerous studies have found that the ability of corneal morphological parameters to diagnose keratoconus is high [23–26]. In the Pentacam corneal topographic map, combining the anterior and posterior surface height map, cornea thickness spatial profile (CTSP) and percentage thickness increase (PTI), constitutes the Belin/Ambrosio Enhanced Ectasia Display (Belin-Ambrósio deviation [BAD-D]) software. BAD-D in this software is calculated based on a regression analysis that weights differently the parameters, which can effectively diagnose keratoconus [27]. Renato Ambrósio et al. [27] found that the AUC of BAD-D in detecting keratoconus was 1.00, with a critical value of 2.11, sensitivity of 1, and specificity of 1. In this study, BAD-D could effectively detect keratoconus, which was similar to previous reports [26]. In addition, BAD-D could effectively diagnose keratoconus at different stages (all AUC > 0.96). The AUC of BAD-D in distinguishing stage I keratoconus from normal cornea was 0.963, with a critical value of 1.595, and sensitivity and specificity of 1 respectively. Stage I keratoconus represents a relatively early stage of the disease, suggesting that BAD-D can be effective in the diagnosis of early keratoconus; and the diagnostic efficiency of BAD-D increases with the increase in keratoconus stages.

Progression of keratoconus leads to the destruction of corneal stroma structure, causing instability of corneal biomechanical properties and weakening of mechanical strength [4]. Roberts and Dupps suggested that the underlying cause of corneal ectasia or keratoconus is the abnormal biomechanical properties of the cornea, while the morphological changes of the cornea are secondary manifestations [28]. With the recent development of corneal biomechanics in vivo, measuring the biomechanical properties of keratoconus has become a hotspot in ophthalmology.

The current study found significant differences in most biomechanical parameters between the normal cornea and keratoconus as well as between the normal cornea and keratoconus at different stages. During the first and second applanation status, the applanation time and length of the keratoconus group were shorter than those of the normal group, and the applanation velocity was greater than that of the normal group. The easier deformation in the keratoconus group was due to the weaker matrical collagen fibers and thinner cornea. The results showed that HC DfL, HC DfA and PD of the keratoconus group were larger than those of the normal group, while the central curvature radius of the keratoconus group was smaller than that of the normal group. These findings indicated that the mechanical strength of cornea is weakened in keratoconus, leading to increased deformation amplitude (DA) under the same external force.

Previous biomechanical studies found that the maximum DA was the most effective parameter in diagnosing keratoconus, with an AUC of 0.882, but there was significant overlap between the keratoconus group and the control group [10]. Salomao et al. found significant statistical differences in corneal biomechanical parameters between the normal cornea and keratoconus, but the AUCs of the parameters overlapped between 0.673 and 0.852 [29]. With the update of Corvis ST software, new biomechanical parameters have been proposed such as DA Ratio 2, Integrated Radius, ARTh, SPA1 and CBI. CBI is a combination of dynamic corneal deformation parameters such as DA ratio, A1V, ARTh and SPA1. Studies have shown that some new parameters have high efficiency in the diagnosis of keratoconus [30, 31]. In this study, these new parameters could effectively diagnose keratoconus (all AUC > 0.9), which was similar to previous reports [30]. According to DeLong curve analysis, DA Ratio 2, Integrated Radius and SPA1 had higher diagnostic efficiency than ARTh and CBI. In contrast, Pratik et al. found that CBI was more efficient than SPA1 in diagnosing keratoconus [14]. Sedaghat et al. [32] also found that the AUC of CBI was higher than other biomechanical parameters. This difference in our findings may be due to the included sample size or differences in the degree of keratoconus. In the present study, DA Ratio 2, Integrated Radius, ARTh, SPA1 and CBI had relatively low efficiency in distinguishing normal and stage I keratoconus, with ARTh and CBI showing the lowest efficiency in diagnosing stage I keratoconus. Among them, the AUC of CBI in diagnosing stage I keratoconus was 0.761, indicating that the ability of CBI to diagnose early keratoconus was relatively low, which was similar to the report of Pratik et al. [10]. However, with the increase in keratoconus stages, the diagnostic efficiency of these biomechanical parameters also increased, and the efficiency of each parameter in the diagnosis was same in keratoconus III or IV stages.

Recently, Ambrosio et al. used artificial intelligence methods, including random forest analysis and combining corneal morphological parameters of Pentacam with biomechanical parameters of Corvis to develop a new parameter—TBI, which was applied in keratoconus study [13]. They found that TBI had higher diagnostic efficiency (AUC = 1) than parameters obtained with Pentacam and Corvis ST alone in the detection of corneal ectasia, with a critical value of 0.79, and the sensitivity and specificity of 1, respectively. In this study, TBI could effectively distinguish keratoconus from normal cornea (AUC = 0.993), with a critical value of 0.515, and sensitivity of 0.967, and specificity of 1. This difference from the previous threshold may be due to the different degree of keratoconus among the enrolled patients. In graded keratoconus, the efficiency of TBI in the diagnosis of keratoconus in each stage was high (AUC > 0.97). Among them, the AUC of TBI for differentiating normal cornea and stage I keratoconus was 0.979, indicating that TBI could effectively diagnose early keratoconus. Finally, according to DeLong curve analysis, the efficiency of TBI in diagnosing keratoconus in each stage was the same. In this study, according to DeLong curve analysis, BAD-D and TBI had the same efficiency in diagnosing keratoconus, which was higher than other parameters. In contrast, Salomao et al. [33] found that the efficiency of TBI in diagnosing keratoconus was higher than BAD-D. Moreover, the diagnostic efficiency of TBI was the same for keratoconus at all stages.

This study included a large sample size, divided keratoconus into different grades for analysis, and focused on the diagnostic ability of some new parameters for different stages of keratoconus. The limitations of this study were that it was a cross-sectional study, and the enrolled patients were not followed-up. In addition, forme fruste keratoconus was not included in this study; further studies should be conducted in order to explore methods for the diagnosis of more early keratoconus.

In summary, the morphological and biomechanical parameters of cornea in this study could effectively distinguish normal cornea from keratoconus, with BAD-D and TBI being the most efficient parameters. BAD-D and TBI showed high efficiency in the diagnosis of keratoconus of different grades, and the diagnostic efficiency of TBI was the same for keratoconus at all stages. Except for TBI, the efficiency of other parameters in diagnosing keratoconus increased with the increase in keratoconus stages.

## Data Availability

The data that support the findings of this study are available from the corresponding author upon reasonable request.

## Declarations

### Informed consents

Signed written informed consents were obtained from the patients and/or guardians.
